# Exosomes Isolated From Bone Marrow Mesenchymal Stem Cells Exert a Protective Effect on Osteoarthritis via lncRNA LYRM4-AS1-GRPR-miR-6515-5p

**DOI:** 10.3389/fcell.2021.644380

**Published:** 2021-05-28

**Authors:** Xiuhui Wang, Zhuokai Li, Yin Cui, Xu Cui, Cheng Chen, Zhe Wang

**Affiliations:** ^1^Department of Orthopedics, Shanghai University of Medicine and Health Sciences Affiliated Zhoupu Hospital, Shanghai, China; ^2^Department of Orthopedics, Zhongshan Hospital, Fudan University, Shanghai, China

**Keywords:** osteoarthritis, exosomes, GRPR, miR-6515-5p, lncRNA LYRM4-AS1, IL-1β-induced chondrocytes

## Abstract

**Objectives:**

The aim of this study was to investigate the effects of exosomes isolated from human bone marrow mesenchymal stem cells (BMSCs) on osteoarthritis (OA) and a competitive endogenous RNA (ceRNA) network.

**Methods:**

Exosomes were isolated from human BMSCs and characterized by transmission electron microscopy (TEM), Nanosight (NTA), and western blotting. Chondrocytes were treated with interleukin-1β (IL-1β) and then transfected with exosomes. Cell viability and apoptosis were determined using Cell Counting Kit-8 (CCK-8) and flow cytometry, respectively. Cells with IL-1β and exosomes were sequenced, and differentially expressed lncRNAs (DE-lncRNAs) and miRNAs (DE-miRNAs) were identified. Thereafter, a ceRNA network (LYRM4-AS1-GRPR-miR-6515-5p) was chosen for further validation.

**Results:**

TEM, NTA, and western blotting showed that exosomes were successfully isolated, and PKH67 staining showed that exosomes could be taken up by IL-1β-induced chondrocytes. Compared with the control group, IL-1β significantly decreased cell viability and promoted apoptosis (*P* < 0.05), while exosomes reversed the changes induced by IL-1β. For MMP3, AKT, and GRPR, IL-1β upregulated their expression, while exosomes downregulated their expression. For PTEN, there was no significant difference in PTEN expression between the control and IL-1β groups; however, exosomes markedly upregulated PTEN expression. By sequencing, 907 DE-lncRNAs and 25 DE-miRNAs were identified, and a ceRNA network was constructed. The dual-luciferase reporter gene indicated that LYRM4-AS1, miR-6515-5, and GRPR interacted with each other. The results of cell experiments showed that LYRM4-AS1 regulated the growth of IL-1β-induced chondrocytes by GRPR/miR-6515-5p.

**Conclusion:**

Exosomes may alleviate OA inflammation by regulating the LYRM4-AS1/GRPR/miR-6515-5p signaling pathway.

## Introduction

Osteoarthritis (OA) is a painful joint disease characterized by progressive breakdown of articular cartilage, sclerosis of the subchondral bone, and abnormal bone growth (osteophyte) (Guilak et al., 2018). OA remains a major global public health problem causing increasing morbidity and disability and affecting approximately 240 million people worldwide, particularly adults over the age of 45 (Jonsson et al., 2016; Lankhorst et al., 2017; Nelson, 2018). The pathogenesis of OA is related to many factors, such as aging, inflammation, obesity, trauma, and heredity, but its basic molecular mechanisms are still not fully understood. At present, the standard drugs for OA treatment are mainly non-steroidal anti-inflammatory drugs (NSAIDs), opioids, and steroid injections, which can alleviate the symptoms of OA (Poulet and Staines, 2016). However, long-term use of these drugs may lead to a series of adverse effects, including gastrointestinal, renal, and cardiovascular complications (Benyamin et al., 2008; Harirforoosh et al., 2013). Therefore, the discovery of novel pharmacological and physiological pathways may provide potential targets for new drugs to improve the management of OA.

Bone marrow mesenchymal stem cells (BMSCs), a type of pluripotent cell, are the most commonly used stem cells in cell therapy and tissue repair (Fu et al., 2019). Previous studies have shown that BMSC transplantation can facilitate cartilage repair and wound and fracture healing (Chen et al., 2016; Wang et al., 2018), and has significant therapeutic effects on renal damage, articular cartilage injury, and myocardial damage (Zhang et al., 2015; Li L. et al., 2018; Yang et al., 2018). In addition, increasing evidence has indicated that BMSCs can produce and release a series of bioactive factors during the repair process, such as chemokines, cytokines, and growth factors, which directly stimulate target cells to emit functional active mediators in a paracrine manner to play their protective and endogenous regenerative roles (Francois et al., 2013; Oh et al., 2018). However, there are some limitations in BMSC transplantation, such as the survival time of transplanted cells *in vivo* and the immune rejection reaction after transplantation.

Recently, exosomes, as media for cell-to-cell communication, have attracted increasing attention. Exosomes are small (30–300 nm) carriers of proteins, lipids, and nucleic acids (DNA and RNA) (Qin and Xu, 2014). Exosomes isolated from BMSCs have been reported to display prominent and typical functions in BMSCs (Baglio et al., 2015). He et al. (2020) showed that BMSC-derived exosomes could effectively promote cartilage repair, extracellular matrix synthesis, and knee pain relief in an OA rat model. Another study reported that lncRNA PVT1 in BMSC-derived exosomes promoted osteosarcoma growth and metastasis via miR-183-5p/ERG (Zhao et al., 2019). LncRNAs have been reported to play important roles in many biological processes, including proliferation, apoptosis, metabolism, and metastasis (Qu et al., 2016; Li X. et al., 2018). Additionally, lncRNAS can serve as a competitive endogenous RNA (ceRNA) to facilitate mRNA expression by sponging microRNAs (miRNAs). A study by Li Z. et al. (2019) showed that exosomal ZFAS1 knockdown induced apoptosis in esophageal squamous cell carcinoma cells and suppressed their proliferation and invasion via the STAT3/miR-124 axis. In MSCs, exosomal lncRNAs have also been reported as novel regulators of osteogenesis (Tye et al., 2018). However, the specific mechanisms of lncRNAs in OA remain unclear, and ceRNA networks in OA have not been fully elucidated.

In addition, chondrocytes play an important role in the development of OA. Inflammation and inflammatory cytokines are closely associated with the occurrence and progression of OA (Goldring and Otero, 2011). Interleukin-1β (IL-1β), a pro-inflammatory cytokine, has been reported to have a potential effect on the destruction of articular cartilage and has been used to establish the inflammatory environment of OA (Feng et al., 2017). Therefore, in this study, IL-1β was used to treat chondrocytes to construct an OA cell model, and the effects of exosomes isolated from human BMSCs on OA were explored. Subsequently, inflammatory chondrocytes and chondrocytes treated with exosomes were sequenced, and a ceRNA network was selected for further verification. These findings provide novel therapeutic targets and pathways for treating OA.

## Materials and Methods

### Culture of Chondrocytes

Chondrocytes were purchased from CHI Scientific Inc. (Jiangsu, China). The chondrocytes were cultured in DMEM/F12 medium containing 10% fetal bovine serum (FBS, Gibco, Grand Island, NY, United States), 100 U/mL penicillin (Gibco), and 100 μg/mL streptomycin (Gibco), and maintained in an incubator with 5% CO_2_ at 37°C. The chondrocytes were passaged upon reaching 80–90% confluency.

The chondrocytes were identified by type II collagen immunohistochemistry staining. Briefly, the chondrocytes were fixed with 4% paraformaldehyde for 20 min, and then were incubated with 0.5% Triton X-100 (Sigma-Aldrich, United States) for 15 min. After that, 3% H_2_O_2_ was added, and incubated for 15 min. After washing with PBS, the chondrocytes were incubated with anti-collagen II antibody (1:100, Proteintech, Chicago, United States) overnight. Afterward, the cells were treated with HRP-labeled secondary antibody (1:2000, Jackson ImmunoResearch Laboratories, Inc.) for 1 h. After washing, the cells were stained with diaminobenzidine (DAB, Beyotime Biotechnology) for 5 min, and then were redyed with hematoxylin (Sigma-Aldrich). Finally, the chondrocytes were observed under a microscope under a 100 × magnification.

### Isolation and Identification of Exosomes From BMSCs

Human BMSCs were purchased from Cyagen Biosciences Inc. (Guangzhou, China). Human BMSCs were cultured in α-MEM (Gibco) with 5% UltraGRO-Advanced (Gibco) and incubated for 48 h. The cell supernatant (100 mL, approximately 10^7^ cells) was collected for exosome isolation. Exosomes were isolated from the BMSC supernatant by differential centrifugation at 4°C (Bobrie et al., 2012). Briefly, the obtained BMSC supernatant was centrifuged at 300 *g* for 10 min, and then the supernatant was transferred to a new tube. After centrifugation at 10,000 *g* for 30 min, the supernatant was transferred to a new tube and centrifuged at 100,000 *g* for 70 min. The sediment was resuspended in 1 mL PBS and then centrifuged at 10,000 *g* for 60 min. The exosomes were resuspended in 200 μL PBS and kept either at −80°C for long-term preservation or at −20°C for short-term preservation.

The concentration of exosomes was measured using a BCA Protein Concentration Assay Kit (BOSTER) following the manufacturer’s protocol. Based on the method of Zhu et al. (2018), the morphology and ultrastructure of the exosomes were visualized using transmission electron microscopy (TEM, JEOL Ltd., United States). Exosome size distribution was determined using a Nanosight NS300 particle size analyzer (NTA; Malvern Panalytical, Malvern, UK) as described by the method of Soares Martins et al. (2018). Additionally, according to the method described by Yin et al. (2019), the protein levels of CD63, CD9, and HSP70, which are specific proteins of exosomes, were evaluated by western blotting with anti-CD63 antibody (1:2,000, ABclonal), anti-CD9 antibody (1:2,000, Abcam), and anti-HSP70 antibody (1:2,000, Proteintech).

### Establishment of a Chondrocyte Inflammatory Model

Chondrocytes were seeded in a 6-well plate. After the cells adhered to the plate, they were treated with 10 ng/mL IL-1β for 24 h. Then, the inflammatory chondrocytes were treated with different concentrations of exosomes (0, 1, 5, 10, 20, and 50 μg/mL). Cells without treatment served as blank controls. After culturing for 24, 48, and 72 h, cell viability and cell apoptosis were assessed.

### Cellular Uptake of Exosomes in Chondrocytes

Cellular uptake of exosomes in chondrocytes was explored by labeling with PKH67 (green fluorescent cell linker for general cell membrane labeling) using a commercial kit (PKH67GL-1KT; Sigma-Aldrich, United States), according to the manufacturer’s instructions. Briefly, Diluent C (200 μL) was added to the exosomes, and then 300 μL of Diluent C and 4 μL PKH67 dye were added. The mixture was incubated at room temperature for 5 min. Subsequently, 1% bovine serum albumin (600 μL BSA; Sigma-Aldrich, United States) was added to bind excess dye. The mixture was centrifuged at 120,000 *g* for 60 min, and the sediment (PKH67-labeled exosomes) was resuspended in PBS for use.

The chondrocytes were seeded into a 24-well plate at a density of 1 × 10^4^ cells/well and cultured overnight. Next, 10 ng/mL IL-1β was added to the cells for 24 h, and then 10 μg/mL PKH67-labeled exosomes were added. After incubation for 24 h, the chondrocytes were fixed with 4% paraformaldehyde for 15 min. After washing, the cells were stained with 4, 6-diamidino-2-phenylindole (DAPI) and observed under a laser scanning confocal microscope (TCS SP8, Leica Microsystems, Inc., United States) at a magnification of 400×.

### Whole Transcriptome Sequencing

Inflammatory cells treated with PBS and 20 μg/mL exosomes were used as the IL-1β and IL-1β + Exos groups. The chondrocytes in the IL-1β and IL-1β + Exos groups were sequenced by Yanzai Biotechnology (Shanghai) Co., Ltd. (Shanghai, China), as previously described (Park and Kim, 2016). The total RNA was extracted from the cells with different treatments using mirVana^TM^ miRNA Isolation Kit (Thermo Fisher Scientific), and used for whole transcriptome sequencing. DESeq was used to identify the differentially expressed genes (DEGs) between inflammatory cells and inflammatory cells treated with exosomes, including differentially expressed lncRNAs (DE-lncRNAs) and differentially expressed miRNAs (DE-miRNAs). The thresholds for screening DE-lncRNAs and DE-miRNAs were log_2_Fold change (FC) > 2, *P*-value < 0.05, and log_2_Fold change (FC) > 1, *P*-value < 0.05, respectively. Gene Ontology (GO) and Kyoto Encyclopedia of Genes and Genomes (KEGG) pathway analyses were performed on these DE-lncRNAs and DE-miRNAs. Next, ceRNA networks were analyzed, and a ceRNA network (ENST00000663198.1 [LYRM4-AS1]—ENSG00000126010 (GRPR)—hsa-miR-6515-5p) was selected for further study. Additionally, six DE-lncRNAs (three upregulated and downregulated) were chosen for real-time quantitative PCR (RT-qPCR) verification, and the sequences of the six DE-lncRNAs are shown in Table 1.

**TABLE 1:** The sequences of all primers.

**Primer**		**Sequence (5′–3′)**
ENST00000652428 (LINC00310-209)	F	CCGTGGAATGTCTTTGGC
	R	TGCGTGCTGGAGGATGAA
ENST00000622968 (AC118344.2-201)	F	TCTATTCTGCTGCTCCATT
	R	CGCCCTCGTATCTTGTAT
ENST00000654466.1 (AC025171.1-205)	F	TCGCAAACAGCATTACAT
	R	TTTCCAAGTTCCGAAGAG
ENST00000572856.1 (DLGAP1-AS2-201)	F	CACAGGCTACCACCACTC
	R	CTTCTTCATGCACGCTCT
ENST00000656992.1 (UBA6-AS1-209)	F	CATACTGCCCAAGATAAT
	R	CTCTGTTCTGTTCCGTTG
ENST00000626826.1 (HELLPAR-201)	F	CAGCCCTCTACTTACCCA
	R	TGTCAAGTGTTCTTACGG
ENST00000663198.1 (LYRM4-AS1)	F	CCCAACTGTAACGACCAC
	R	TTAACATCAAACTAAGGCAC
IL-1β	F	ATGATGGCTTATTACAGTGGCAA
	R	GTCGGAGATTCGTAGCTGGA
IL-6	F	CCTGAACCTTCCAAAGATGGC
	R	TTCACCAGGCAAGTCTCCTCA
GAPDH	F	TGACAACTTTGGTATCGTGGAAGG
	R	AGGCAGGGATGATGTTCTGGAGAG
miR-6515-5p	F	GCCGTTGGAGGGTGTGGAA
	R	GTCGTATCCAGTGCAGGGTCCGAG
		GTATTCGCACTGGATACGACGATGTC
U6	F	CTCGCTTCGGCAGCACA
	R	AACGCTTCACGAATTTGCGT

### Dual-Luciferase Reporter Gene Assay

The sequences of LYRM4-AS1, hsa-miR-6515-5p mimics, and GRPR 3-untranslated region (3-UTR) were synthesized by Yanzai Biotechnology (Shanghai) Co., Ltd. (Shanghai, China). The psiCHECK2 vector (Yanzai Biotechnology (Shanghai) Co., Ltd.) and pGL3-basic vector (Yanzai Biotechnology (Shanghai) Co., Ltd.) were used to construct the psiCHECK2-LYRM4-AS1 reporter plasmid (psiCHECK2-LYRM4-AS1) and the 3′-UTR GRPR reporter plasmid (pGL3-GRPR). Next, psiCHECK2 (500 ng) or psiCHECK2-LYRM4-AS1 (500 ng), and pGL3-basic vector (500 ng) or pGL3-GRPR (500 ng) were co-transferred into 293T cells with has-miR-6515-5p mimics (100 nM) or negative control (NC) mimics (100 nM) using Lipofectamine 3000 (Thermo Fisher Scientific) following the manufacturer’s recommendations. A dual luciferase reporter system (Promega, Madison, WI, United States) was used to detect luciferase reactivity.

### Cell Transfection

Small interfering (si)-lncRNA LYRM4-AS1 or si-negative control (NC), and miR-6515-5p inhibitor or NC inhibitor were prepared and purchased from Yanzai Biotechnology Co., Ltd. (Shanghai, China). Cell transfection was performed as described previously (Lin et al., 2017). Briefly, chondrocytes were seeded in a 24-well plate. After the cells reached a density of 70%, the cell culture medium was changed to serum-free medium, and the cells were transfected with 100 nM si-lncRNA LYRM4-AS1 or si-NC, and 100 nM miR-6515-5p inhibitor or NC inhibitor using Lipofectamine 3000 (Thermo Fisher Scientific), according to the manufacturer’s instructions. After transfection for 6 h, the medium was replaced with complete medium, and IL-1β (10 ng/mL) was added to the cells. After treatment for another 48 h, total RNA of the cells with different treatments was isolated, and the expression levels of lncRNA LYRM4-AS1 and miR-6515-5p were determined using RT-qPCR to assess the transfection efficiency. The primer sequences of the lncRNA LYRM4-AS1 and miR-6515-5p are shown in Table 1.

### Cell Viability and Cell Apoptosis Assays

Cell viability of the chondrocytes with different treatments was measured using the Cell Counting Kit-8 (CCK-8, Beyotime Biotechnology, Shanghai, China) following the manufacturer’s protocols. The cells were treated with different treatments, and 10 μL of CCK-8 was added. After incubation for 2 h, the absorbance was measured at 450 nm using a microplate reader (Multiskan MK3; Thermo Fisher Scientific).

The Annexin V-FITC/PI apoptosis assay kit (Beyotime Biotechnology) was used to assess cell apoptosis of the chondrocytes with different treatments in accordance with the manufacturer’s recommendations. The cells were harvested and centrifuged at 1000 *g* for 5 min. After washing with PBS, the cells were resuspended in 1 × binding buffer (100 μL). Next, 5 μL of FITC-Annexin V and 5 μL of PI (50 μg/mL) were added. After incubation at 25°C in the dark for 15 min, 400 μL of 1 × binding buffer was added, and the images were acquired by flow cytometry. The apoptosis rate was calculated using CellQuest software (Becton, Dickinson and Company, NJ, United States).

### RT-qPCR

Total RNA was extracted from the cells with different treatments using TRIzol reagent (Thermo Fisher Scientific) and 2 times volume of isopropanol for RNA precipitate (Thermo Fisher Scientific) according to the manufacturer’s instructions. The purity and concentration of the total RNA were evaluated using a microplate reader. Stem ring method was used to determine the levels of miR-6515-5p (Wang et al., 2021), and *U6* was served as a reference gene. Total RNA was reverse transcribed into cDNA using a cDNA Reverse Transcription Kit (Takara Bio Inc.) according to the manufacturer’s protocol. The RT-qPCR reaction conditions were as follows: pre-denaturation at 95°C for 3 min; 40 cycles at 95°C for 10 s and 60°C for 30 s; melt curve at 60–95°C with an increment of 0.5°C for 10 s. The sequences of all primers are listed in Table 1. Glyceraldehyde-3-phosphate dehydrogenase (GAPDH) served as a housekeeping gene, and the relative mRNA expression of Interleukin-1β (*IL-1*β) and *IL-6* was calculated using the 2^–ΔΔCt^ method (Xu et al., 2019).

### Western Blot

Total protein was isolated from the chondrocytes with different treatments using RIPA protein lysis buffer, and protein concentrations were determined using a BCA assay kit (BOSTER). The protein samples (20 μg) were separated by 10% SDS-PAGE and then transferred to PVDF membranes. After blocking with 5% skim milk at 37°C for 2 h, the membranes were incubated with anti-matrix metallopeptidase 13 (MMP13) antibody (1:4,000; Proteintech Group, Inc., Rosemont, IL, United States), anti-AKT serine/threonine kinase 1 (AKT1) antibody (1:2,000, Proteintech Group, Inc.), anti-phosphatase and tensin homolog (PTEN) antibody (1:4,000, Bioss), anti-gastrin-releasing peptide receptor (GRPR) antibody (1:2,000, Abcam), and anti-GAPDH antibody (1:2,000, Abcam) at 4°C overnight. The membranes were then incubated with the secondary antibody (goat anti-rabbit IgG-HRP, 1:1,000; Jackson ImmunoResearch Laboratories Inc., PA, United States). After incubation at 37°C for 2 h, the Millipore ECL system (Shanghai Tanon Technology Co., Ltd., Shanghai, China) was used to visualize the protein bands.

### Statistical Analysis

Data are expressed as the mean ± standard deviation (SD). GraphPad Prism 5 (GraphPad Software, San Diego, CA) was used to perform all statistical analyses. For the comparison of two groups, Student’s *t*-test was used. One-way analysis of variance (ANOVA) and Tukey’s test were used to compare the differences between more than two groups. Statistical significance was set at *p* < 0.05.

## Results

### Identification of Exosomes Isolated Form Human BMSCs

**FIGURE 1:**
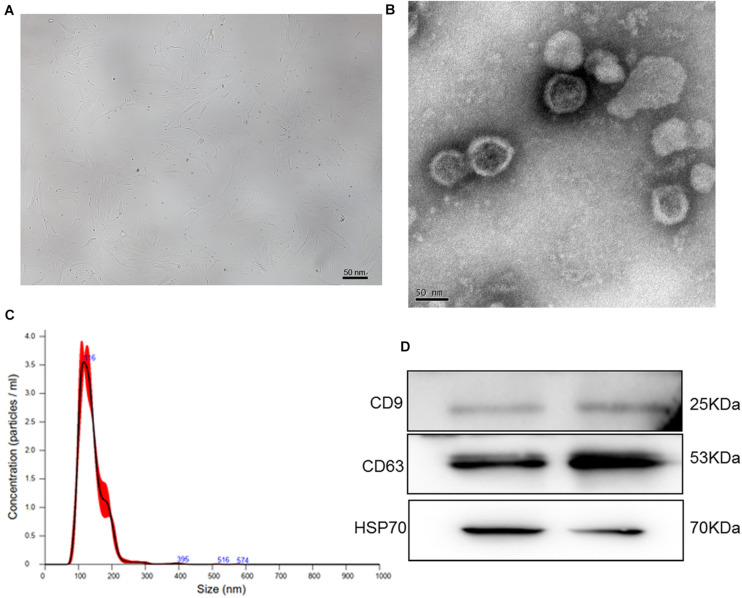
**(A)** The morphology of human bone marrow mesenchymal stem cells (BMSCs). **(B)** Transmission electron microscopy image of exosomes isolated from human BMSCs. **(C)** The size distribution of exosomes determined by nanoparticle tracking analysis. **(D)** The images of exosome-specific CD9, CD63, and HSP70 proteins examined by western blot.

To isolate exosomes from human BMSCs, human BMSCs were cultured for the first time. After the cells reached 80% confluence, BMSCs displayed proliferation ability and showed a relatively homogeneous population of spindle cells (Figure 1A). Thereafter, exosomes were extracted from human BMSCs and identified by TEM, NTA, and western blotting. TEM showed that exosomes exhibited a cup-shaped or nearly round morphology with a diameter of approximately 100 nm (Figure 1B). NTA revealed that the major peak of particle size was approximately 116 nm (Figure 1C), which was in accordance with the size distribution of exosomes reported previously (Tkach and Thery, 2016). Furthermore, western blotting showed that CD9, CD63, and HSP70, which are exosome markers, were expressed in the exosomes (Figure 1D). These results indicate that exosomes were successfully isolated from human BMSCs using the ultracentrifugation method.

### Establishment of a Chondrocyte Inflammatory Model and Cellular Uptake of Exosomes

**FIGURE 2:**
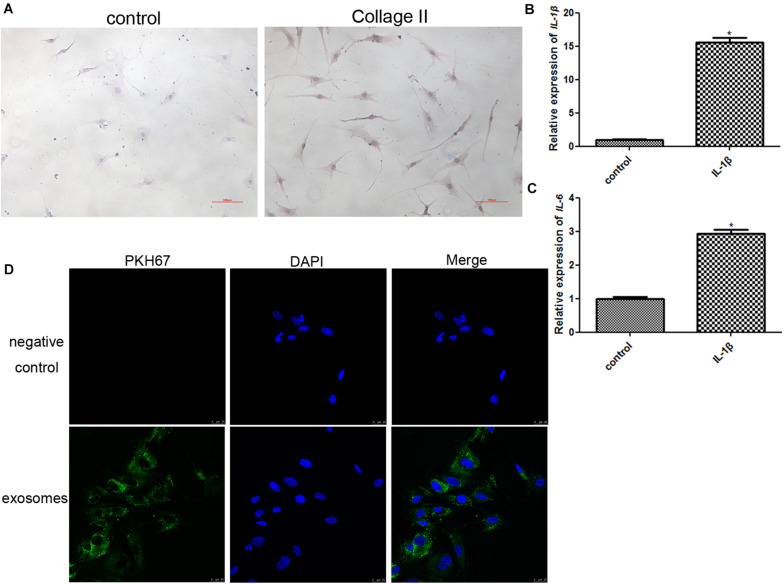
**(A)** Identification of chondrocytes using immunocytochemical staining of collagen II (100×). **(B)** The expression level of IL-1β after chondrocytes were induced by IL-1β. *P < 0.05 vs. control group. *n* = 3. **(C)** The expression level of IL-6 after chondrocytes were induced by IL-1β. *P < 0.05 vs. control group. *n* = 3. **(D)** PKH67-labeled exosomes could be taken up by chondrocytes induced by IL-1β after co-culture.

In order to identify the chondrocytes, the expression of collagen II was determined. As shown in Figure 2A, type II collagen immunocytochemical staining confirmed the cellular phenotype of obtained chondrocytes, and showed that the cells were stained dark yellow-brown. Therefore, the positive results of collagen II staining replied that the obtained cells were chondrocytes.

The chondrocytes were treated with 10 ng/mL IL-1β for 24 h to construct an inflammatory cell model, and the expression of IL-1β and IL6 was determined to assess the model. After IL-1β treatment, the expression of IL-1β and IL6 was significantly upregulated compared to that in the control group (*P* < 0.05, Figures 2B,C). These results implied that inflammatory chondrocytes were successfully established by treatment with IL-1β.

In addition, PKH67 was used to label exosomes (green fluorescence), and PKH67-labeled exosomes were co-cultured with inflammatory chondrocytes for 24 h. After co-culture, most inflammatory chondrocytes displayed intracellular green fluorescence (Figure 2D). These results indicated that exosomes isolated from human BMSCs could be taken up by chondrocytes induced by IL-1β.

### The Effects of Exosomes on the Growth of Chondrocytes Treated With IL-1β and on the Expression of MMP13, AKT1, PTEN, and GRPR

**FIGURE 3:**
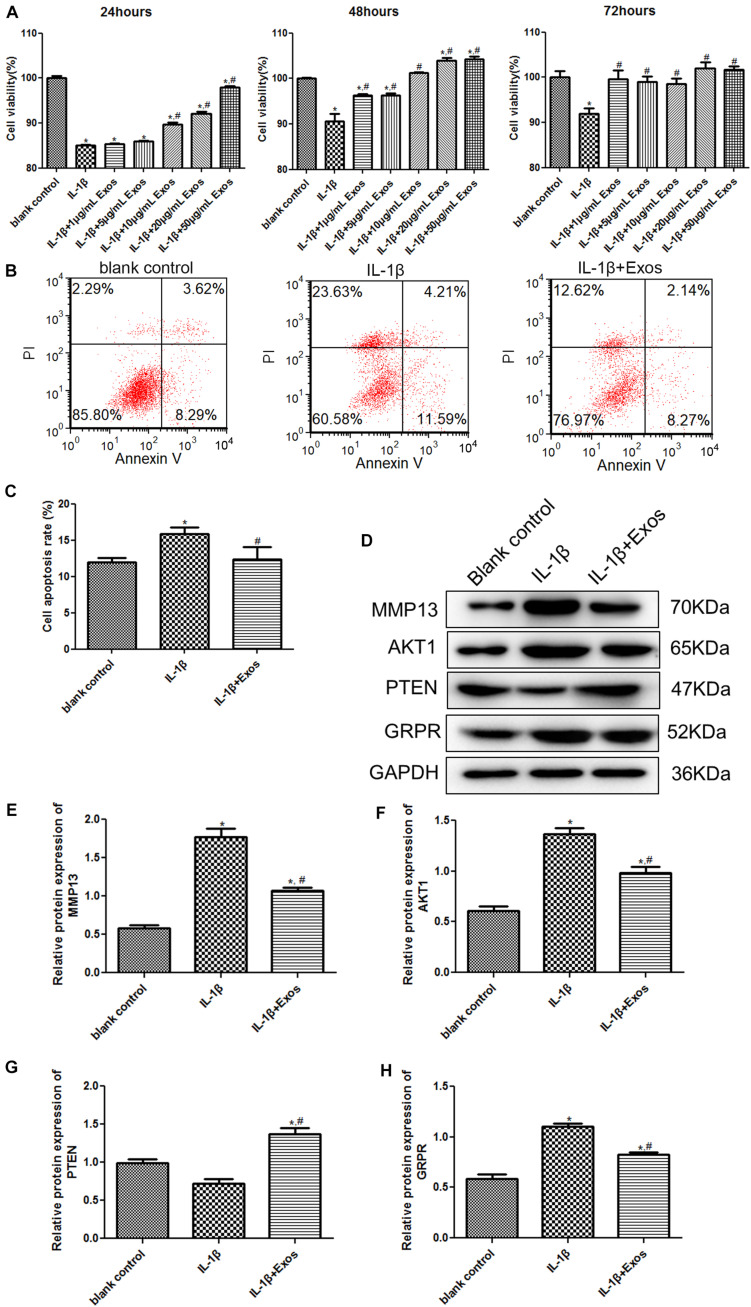
**(A)** Cell viability of chondrocytes treated with different concentrations of exosomes for 24, 48, and 72 h using the Cell Counting Kit-8 (CCK-8) assay. *n* = 3. **(B)** The images of cell apoptosis acquired by flow cytometry. **(C)** The cell apoptosis rate of chondrocytes treated with exosomes analyzed by CellQuest software. *n* = 3. **(D)** The protein bands acquired by western blot. The protein expressions of MMP13 **(E)**, AKT1 **(F)**, PTEN **(G)**, and GRPR **(H)** after chondrocytes were treated with exosomes determined by western blot. *n* = 3. *P < 0.05 vs. blank control group; ^#^*P* < 0.05 vs. IL-1β group.

To understand the roles of exosomes in chondrocytes, cell viability and apoptosis were determined. After culturing for 24, 48, and 72 h, there was no significant difference in the cell viability between the normal cells and normal cells treated with exosomes (*P* > 0.05, Supplementary Figure 1). Therefore, we explored the effects of exosomes on chondrocytes. Compared with the blank control group, the cell viability of chondrocytes was significantly inhibited after IL-1β treatment (*P* < 0.05, Figure 3A). After treatment with 1 and 5 μg/mL exosomes for 24 h, the cell viability of chondrocytes was similar to that of the IL-1β group (*P* > 0.05), whereas after treatment with 10, 20, and 50 μg/mL exosomes for 24 h, the cell viability was significantly higher than that of the IL-1β group (*P* < 0.05). When the inflammatory cells were treated with different concentrations of exosomes for 48 h, the cell viability was significantly increased compared with the IL-1β group (*P* < 0.05), and when the exosome concentrations were 20 and 50 μg/mL, the cell viability was higher than that in the blank control and IL-1β groups (*P* < 0.05). Therefore, treatment with 20 μg/mL exosomes for 48 h was selected for subsequent experiments. For cell apoptosis, the cell apoptosis rate in the IL-1β group (15.87 ± 0.49%) was markedly higher than that in the blank control group (11.96 ± 0.34%, *P* < 0.05, Figures 3B,C); while after treatment with 20 μg/mL exosomes, the cell apoptosis (12.32 ± 1.02%) was restored to a similar level to that of the blank control group (*P* > 0.05, Figures 3B,C). Taken together, IL-1β treatment suppressed the viability of chondrocytes and promoted their apoptosis, whereas exosomes could properly restore the changes in cell vitality and apoptosis induced by IL-1β.

To further investigate the molecular mechanisms by which exosomes affect cell viability and apoptosis, the expression of MMP13, AKT1, PTEN, and GRPR was examined by western blotting. Compared with the blank control group, the expression of MMP3 and AKT1 was significantly upregulated after IL-1β treatment, while after exosome treatment, their expression was down-regulated compared to the IL-1β group (Figures 3D–F). For PTEN, there was no significant difference in its expression between the blank control group and the IL-1β group (*P* > 0.05); however, its expression after exosome treatment was significantly upregulated (Figures 3D,G). In addition, the change trend of GRPR expression was similar to that of MMP13 and AKT expression (Figures 3D,H).

### Analyses of Whole Transcriptome Sequencing and RT-qPCR Verification

**FIGURE 4:**
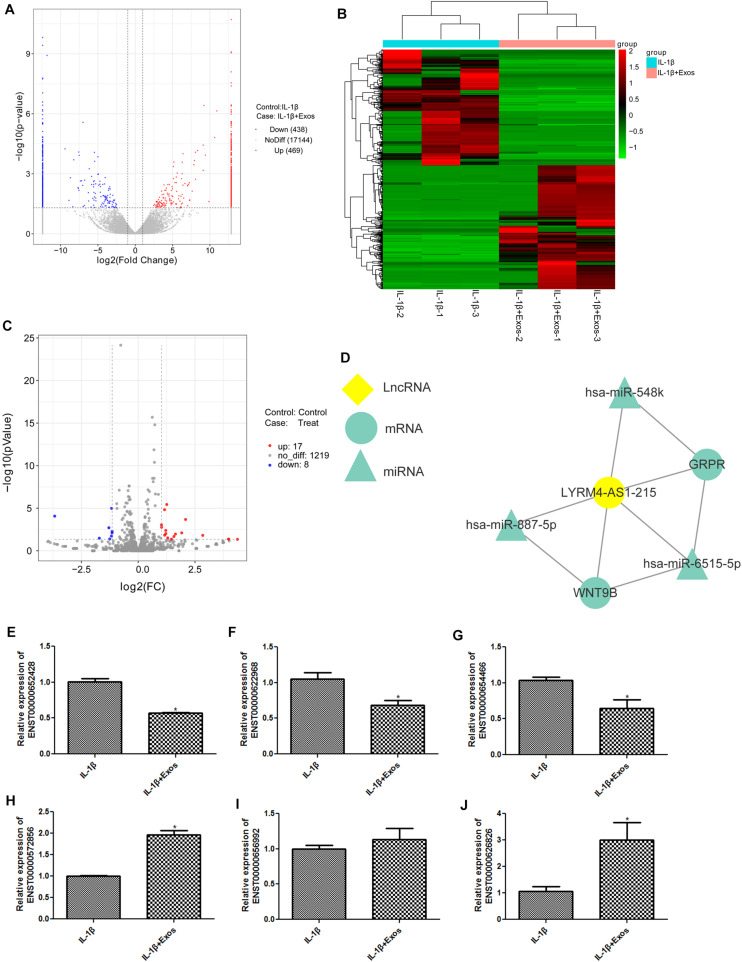
**(A)** The volcano plot of differentially expressed lncRNAs (DE-lncRNAs). **(B)** The heat map of DE-lncRNAs. **(C)** The volcano plot of differentially expressed miRNAs (DE-miRNAs). **(D)** A ceRNA network about lncRNA LYRM4-AS1 was built. **(E)** The expression of lncRNA ENST00000652428. **(F)** The expression of lncRNA ENST00000622968. **(G)** The expression of lncRNA ENST00000654466. **(H)** The expression of lncRNA ENST00000572856. **(I)** The expression of lncRNA ENST00000656992. **(J)** The expression of lncRNA ENST000006268268. *n* = 3. *P < 0.05 vs. IL-1β group.

The cells in the IL-1β and IL-1β + Exos groups were sequenced, and DEGs between inflammatory cells and inflammatory cells treated with exosomes were analyzed. A total of 907 DE-lncRNAs were identified, including 438 downregulated lncRNAs and 469 upregulated lncRNAs (Figure 4A). The heatmap distribution of the DE-lncRNA expression is shown in Figure 4B. GO and KEGG analyses were then performed on the DE-lncRNAs (Supplementary Figures 2A,B). Supplementary Figure 1A displays the top10 GO terms in cellular component (CC), molecular function (MF), and biological process (BP), including membrane-bounded organelle, SMAD2-SMAD3 protein complex and cytoplasm in CC; SMAD binding, RNA polymerase II transcription factor activity, and formate-tetrahydrofolate ligase activity in MF; and import across plasma membrane, amino acid transmembrane import, methionine metabolic process, and osteoblast differentiation in BP. These DE-lncRNAs were also enriched in the relaxin signaling pathway, signaling pathways regulating pluripotency of stem cells, protein digestion and absorption, cell cycle, D-arginine and D-ornithine metabolism, IL-17 signaling pathway, and TGF-beta signaling pathway (Supplementary Figure 2B).

Subsequently, 25 DE-miRNAs were screened with the threshold of log_2_FC > 1 and *P*-value < 0.05, including 17 upregulated and 8 downregulated miRNAs (Figure 4C and Table 2). These DE-miRNAs were associated with the intrinsic components of the plasma membrane, cell, and plasma membrane region in CC, and DNA binding transcription factor activity, ion binding, protein binding in MF, anatomical structure morphogenesis, regulation of primary metabolic processes, regulation of nitrogen compound metabolic processes, regulation of cellular metabolic processes, and nervous system development in BP (Supplementary Figure 1C). Additionally, these DE-miRNAs were related to the MAPK signaling pathway, TNF signaling pathway, terpenoid backbone biosynthesis, Rap1 signaling pathway, cAMP signaling pathway, and calcium signaling pathway (Supplementary Figure 1D).

**TABLE 2:** The differentially expressed miRNAs (DE-miRNAs) between inflammatory cells and inflammatory cells treated with exosomes.

**ID**	**logFC**	***P*-value**	**Regulation**
hsa-miR-4804-5p	4.369282269	0.044934589	Up regulation
hsa-miR-5188	3.985399519	0.043346613	Up regulation
hsa-miR-6802-3p	3.978271057	0.042313985	Up regulation
hsa-miR-124-5p	2.842255887	0.015303299	Up regulation
hsa-miR-3065-5p	2.086145606	0.000207661	Up regulation
hsa-miR-206	1.916945659	0.007803674	Up regulation
hsa-miR-6515-5p	1.639455774	0.010792166	Up regulation
hsa-miR-4504	1.572682075	0.020863067	Up regulation
hsa-miR-449a	1.46759187	0.042832155	Up regulation
hsa-miR-133a-3p	1.30080826	0.030564227	Up regulation
hsa-miR-483-3p	1.259195958	3.6597E-06	Up regulation
hsa-miR-548k	1.217801224	0.009536832	Up regulation
hsa-let-7c-3p	1.214944911	0.004027594	Up regulation
hsa-miR-7976	1.165499887	0.014562738	Up regulation
hsa-miR-585-3p	1.161558169	1.48771E-05	Up regulation
hsa-miR-548e-3p	1.028993871	0.001738362	Up regulation
hsa-miR-4746-5p	1.026294946	0.000885642	Up regulation
hsa-miR-7704	−3.673672131	8.48286E-05	Down regulation
hsa-miR-147a	−1.71092359	0.033242609	Down regulation
hsa-miR-376b-5p	−1.290335472	0.001974939	Down regulation
hsa-miR-191-3p	−1.254283585	0.041073194	Down regulation
hsa-miR-887-5p	−1.174667937	0.018450622	Down regulation
hsa-miR-29c-3p	−1.169956008	1.03332E-05	Down regulation
hsa-miR-376c-5p	−1.149515746	0.007776615	Down regulation
hsa-miR-154-3p	−1.14467181	0.005211388	Down regulation

Based on the expression of DE-lncRNAs, DE-miRNAs, and mRNAs obtained by sequencing, we found that the expression of lncRNA LYRM4-AS1 was in line with the forecast, and then a ceRNA network of lncRNA LYRM4-AS1 was built (Figure 4D). It was found that lncRNA LYRM4-AS1 interacted with miR-887-5p, miR-6515, and miR-548k. Because miR-887-5p was downregulated, miR-6515-5p and miR-548k were considered for further study. A previous study showed that the GRP pathway plays an important role in chronic arthritis (Oliveira et al., 2011). Therefore, we selected the lncRNA LYRM4-AS1—mRNA GRPR—miR-6515-5p for further experimental validation.

In addition, three upregulated DE-lncRNAs (ENST 00000572856, ENST00000656992, and ENST00000626826) and three downregulated DE-lncRNAs (ENST00000652428, ENST00000622968, and ENST00000654466) were selected to determine their expression in inflammatory cells and inflammatory cells following exosome treatment. It is clear that the expression of ENST00000652428, ENST00000622968, and ENST00000654466 was significantly downregulated in the IL-1β + Exos group, whereas the expression of ENST00000572856 and ENST00000626826 were both significantly upregulated in the IL-1β + Exos group, compared with the IL-1β group (*P* < 0.05, Figures 4E–H,J). However, there was no significant difference in ENST00000656992 expression between the two groups (*P* > 0.05, Figure 4I). The results showed that the consistency rate between sequencing analyses and RT-qPCR results was 83.33%, which indicated a high relative reliability of the sequencing results.

### Interaction Among lncRNA LYRM4-AS1, miR-6515-5p, and GRPR

**FIGURE 5:**
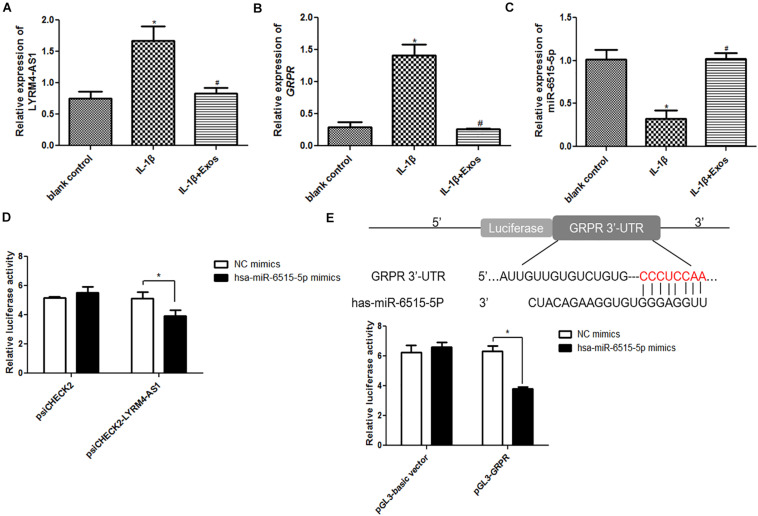
The relative expression of lncRNA LYRM4-AS1 **(A)**, mRNA GRPR **(B)**, and miR-6515-5p **(C)** in the blank control group, IL-1β group, and IL-1β + Exo group. *n* = 3. *P < 0.05 vs. blank control group; ^#^*P* < 0.05 vs. IL-1β group. **(D)** LncRNA LYRM4-AS1 interacted with miR-6515-5p. *n* = 3. *P < 0.05 vs. negative control (NC) mimics. **(E)** GRPR directly binds with miR-6515-5p. *n* = 3. *P < 0.05 vs. NC mimics.

The expression of lncRNA LYRM4-AS1, miR-6515-5p, and GRPR was first determined in the different groups. Compared with the blank control group, lncRNA LYRM4-AS1 and mRNA GRPR expression was significantly upregulated in the IL-1β group (*P* < 0.05), while exosome treatment downregulated their expression and restored their levels to levels similar to those of the blank control group (*P* > 0.05, Figures 5A,B). However, for miR-6515-5p, its expression was significantly down-regulated in the IL-1β group compared to the blank control group (*P* < 0.05), and its expression markedly returned to a level similar to that of the blank control group (*P* > 0.05, Figure 5C). These results indicated that the expression trends of lncRNA LYRM4-AS1, mRNA GRPR, and miR-6515-5p were consistent with the sequencing results.

Next, the interaction between lncRNA LYRM4-AS1, miR-6515-5p, and GRPR was analyzed. There was no significant difference between NC mimics and miR-6515-5p mimics in the psiCHECK2 plasmid (*P* > 0.05), whereas in the psiCHECK2-LYRM4-AS1 plasmid, the relative luciferase activity in the miR-6515-5p mimic group was significantly lower than that in the NC mimics group (*P* < 0.05, Figure 5D). Additionally, in the pGL3-GRPR plasmid, the relative luciferase activity in the miR-6515-5p mimics group was lower than that in the NC mimics group (*P* < 0.05, Figure 5E). These results implied that lncRNA LYRM4-AS1, miR-6515-5, and GRPR mRNA could interact with each other.

### The Effects of lncRNA LYRM4-AS1 on the Growth of Inflammatory Chondrocytes and on the Expression of Related Proteins

**FIGURE 6:**
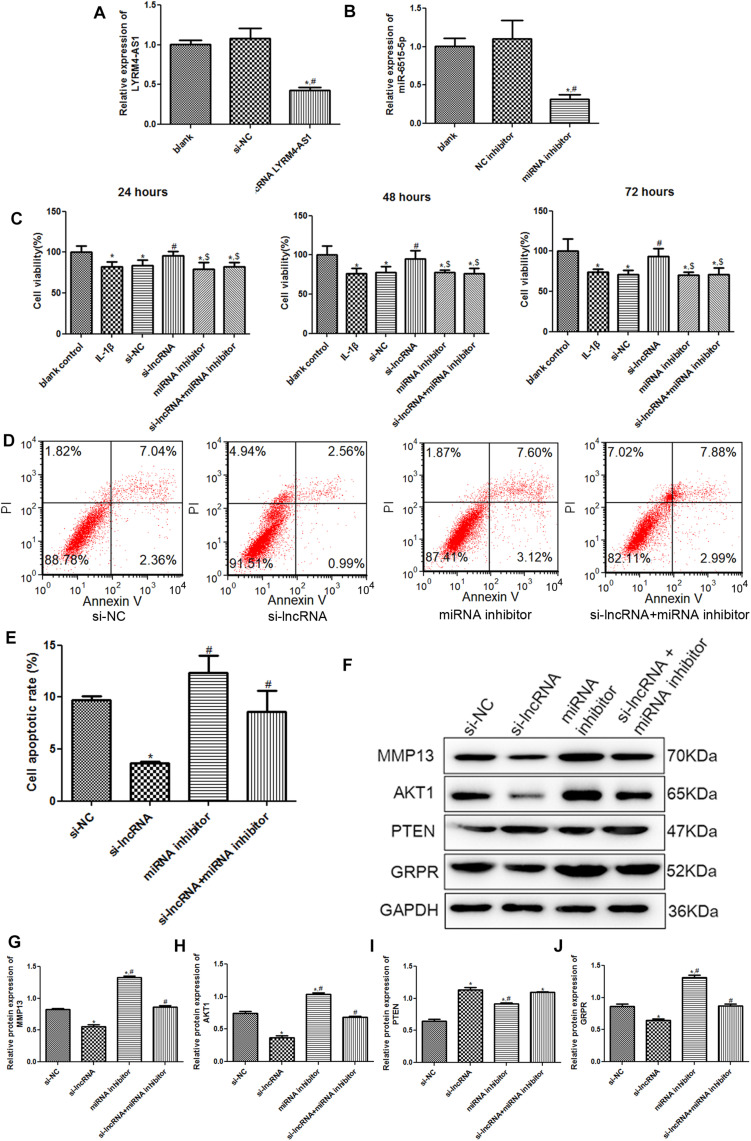
**(A)** The relative expression of lncRNA LYRM4-AS1 after transfection. *n* = 3. *P < 0.05 vs. blank group; ^#^*P* < 0.05 vs. si-NC group. **(B)** The relative expression of miR-6515-5p after transfection. *n* = 3. *P < 0.05 vs. blank group; ^#^*P* < 0.05 vs. NC inhibitor group. **(C)** After cell transfection, the cell viability of chondrocytes was examined using CCK-8 assay. *n* = 3. *P < 0.05 vs. blank control group; ^#^*P* < 0.05 vs. IL-1β group. ^$^*P* < 0.05 vs. si-lncRNA LYRM4-AS1 group. **(D)** The images of cell apoptosis acquired by flow cytometry. **(E)** The cell apoptosis rate of chondrocytes after transfection analyzed by CellQuest software. *n* = 3. *P < 0.05 vs. si-NC group; ^#^*P* < 0.05 vs. si-lncRNA LYRM4-AS1 group. **(F)** The protein bands of MMP13, AKT1, PTEN, and GRPR detected by western blot. The relative protein expression of MMP13 **(G)**, AKT1 **(H)**, PTEN **(I)**, and GRPR **(J)** in different groups. *n* = 3. *P < 0.05 vs. si-NC group; ^#^*P* < 0.05 vs. si-lncRNA group.

To further investigate the roles of lncRNA LYRM4-AS1 in cartilage repair, chondrocytes with lncRNA LYRM4-AS1 interference and miR-6515-5p knockdown were constructed. There was no significant difference in the lncRNA LYRM4-AS1 expression between the blank and si-NC groups (*P* > 0.05); however, after transfection with si-lncRNA LYRM4-AS1, its expression was significantly decreased (*P* < 0.05, Figure 6A). miR-6515-5p expression was markedly downregulated after transfection with miR-6515-5p inhibitor compared to that in the blank and NC inhibitor groups (*P* < 0.05, Figure 6B). These results indicate that chondrocytes with lncRNA LYRM4-AS1 interference and miR-6515-5p knockdown were successfully established.

Subsequently, cell viability and apoptosis were detected. After culturing for 24, 48, and 72 h, there was no significant difference in cell viability between the IL-1β and si-NC groups (*P* > 0.05, Figure 6C). Compared with the blank control group, IL-1β induction significantly inhibited the viability of chondrocytes (*P* < 0.05). When lncRNA LYRM4-AS1 was knocked down, the cell viability of IL-1β-induced chondrocytes was evidently increased compared with that of the IL-1β group (*P* < 0.05), and was restored to a level similar to that of the blank control group (*P* > 0.05). However, in the cells with both lncRNA LYRM4-AS1 and miR-6515-5p knockdown, cell viability was significantly lower than that in the si-lncRNA LYRM4-AS1 group (*P* < 0.05) and was similar to that of the IL-1β group (*P* > 0.05, Figure 6C). Next, apoptosis in different groups was analyzed. Compared with the si-NC group, apoptosis was significantly reduced in the si-lncRNA LYRM4-AS1 group (*P* < 0.05), while in the cells with both lncRNA LYRM4-AS1 and miR-6515-5p knocked down, the cell apoptosis was significantly increased compared with the si-lncRNA LYRM4-AS1 group (*P* < 0.05, Figures 6D,E). These results indicated that lncRNA LYRM4-AS1 interference could enhance cell viability and suppress cell apoptosis in IL-1β-induced chondrocytes, while miR-6515-5p knockdown reversed the changes in cell viability and apoptosis caused by lncRNA LYRM4-AS1 interference.

Finally, the protein expression of MMP13, AKT1, PTEN, and GRPR in different groups was examined by western blotting (Figure 6F). Compared to the si-NC group, lncRNA LYRM4-AS1 interference significantly downregulated the expression of MMP3, AKT1, and GRPR (*P* < 0.05); however, miR-6515-5p knockdown markedly reversed the expression changes of MMP3, AKT1, and GRPR induced by lncRNA LYRM4-AS1 interference (Figures 6G,H,J). The expression of PTEN after lncRNA LYRM4-AS1 interference was significantly higher than that in the si-NC group. After miR-6515-5p knockdown, its expression was also higher than that in the si-NC group but was lower than that in the si-lncRNA group (Figure 6I). In addition, there was no significant difference in PTEN expression between the si-lncRNA and si-lncRNA + miRNA inhibitor groups (*P* > 0.05, Figure 6I).

## Discussion

OA, one of the most prevalent chronic diseases, is a degenerative joint disease involving cartilage and many surrounding tissues, and seriously reduces health and quality of life (Litwic et al., 2013). Since the occurrence and development of OA involves complex molecular mechanisms associated with inflammation and degeneration of articular cartilage, there are still many obstacles in the establishment of improved therapies for OA (Wei and Bai, 2016). In our study, exosomes were successfully isolated from human BMSCs and used to treat IL-1β-induced chondrocytes. It was found that exosome treatment significantly increased the viability of chondrocytes induced by IL-1β and inhibited their apoptosis. Then, the cells in the IL-1β and IL-1β + Exos groups were sequenced, and a total of 907 DE-lncRNAs (438 downregulated and 469 upregulated) and 25 DE-miRNAs (8 downregulated and 17 upregulated) were identified. Afterward, a ceRNA network on lncRNA LYRM4-AS1 was constructed, and lncRNA LYRM4-AS1—mRNA GRPR—miR-6515-5p was chosen for further study. The dual-luciferase reporter gene indicated that LYRM4-AS1, miR-6515-5, and GRPR interacted with each other. Additionally, LYRM4-AS1 knockdown enhanced cell viability and suppressed the apoptosis of IL-1β-induced chondrocytes, while miR-6515-5p inhibition reversed the changes caused by LYRM4-AS1 knockdown. These results suggest that exosomes could exert a protective effect on OA *in vitro*, and LYRM4-AS1/GRPR/miR-6515-5p may be a novel pathway for OA therapy.

Exosomes, a subset of extracellular vesicles generated by all cell types, have been reported to play essential roles in intercellular communication (Meldolesi, 2018). Previous studies have demonstrated the great therapeutic potential of MSC-derived exosomes in joint disease (Li Z. et al., 2018) and the importance of MSC paracrine secretion in a variety of diseases (Linero and Chaparro, 2014). In this study, TEM, NTA, and western blot results showed that exosomes were successfully isolated from human BMSCs, and then exosomes were used to treat IL-1β-induced chondrocytes. It is clear that IL-1β significantly inhibited the viability of chondrocytes and induced cell apoptosis, while exosomes were able to restore the changes in cell vitality and apoptosis induced by IL-1β. A study by Wang et al. (2017) indicated that exosomes isolated from embryonic MSCs could remit OA by balancing the degradation and synthesis of chondrocyte ECM. Another study reported that MSC-derived exosomes could promote cell viability and inhibit cell apoptosis of inflammatory chondrocytes, and may be a novel cell-free therapeutic approach for OA treatment (Qi et al., 2019). Therefore, we speculated that exosomes isolated from BMSCs could protect against OA by regulating cell viability and apoptosis of chondrocytes.

Thereafter, the protein expression levels of MMP3, AKT, GRPR, and PTEN were determined. It was found that the expression of MMP3, AKT, and GRPR was up-regulated after IL-1β induction, and after exosome treatment, the expression was downregulated. Interestingly, there was no significant difference in PTEN expression between the control and IL-1β groups; however, exosomes markedly upregulated PTEN expression. MMP3, a member of the MMP family, has been reported to be highly expressed in OA and rheumatoid arthritis (Tong et al., 2017; Lerner et al., 2018). Zeng et al. (2019) showed that high expression of MMP3 was observed in OA synovial cells, and curcumin could control cell proliferation and induce cell apoptosis by inhibiting MMP3 expression, eventually alleviating OA inflammation. AKT, a serine/threonine protein kinase, is an effector of the PI3K/AKT signaling pathway (Malemud, 2015) and is involved in multiple biological processes, including cell growth, apoptosis, migration, and metabolism (Zhang et al., 2018). PTEN is an important negative regulator of the PI3K/AKT signaling pathway, and loss of PTEN can lead to the activation of the PI3K/AKT signaling pathway (Xi et al., 2020). A study by Zhang et al. (2018) indicated that miRNA-130a could regulate cell growth and relieve OA via the PTEN/PI3K/AKT signaling pathway. Additionally, GRPR is a member of the G-protein coupled receptor superfamily and affects cell growth and invasion (Elshafae et al., 2016). Combined with our results, it can be inferred that exosomes isolated from BMSCs may mediate cell viability and apoptosis induced by IL-1β by downregulating MMP3, AKT, and GRPR expression and upregulating PTEN, thus alleviating OA.

To further investigate the specific molecular mechanisms of exosomes, the cells in the IL-1β and IL-1β + Exos groups were sequenced. A total of 907 DE-lncRNAs, including 438 downregulated and 469 upregulated, and 25 DE-miRNAs, including 8 downregulated and 17 upregulated, were identified. These DE-lncRNAs were involved in SMAD binding, osteoblast differentiation, relaxin signaling pathway, IL-17 signaling pathway, and TGF-beta signaling pathway. Additionally, these DE-miRNAs were enriched in the regulation of nitrogen compound metabolic process, MAPK signaling pathway, TNF signaling pathway, Rap1 signaling pathway, cAMP signaling pathway, and calcium signaling pathway. A previous study has shown that SMAD/TGF-β signaling plays an important role in inflammation in temporomandibular joint OA (Li W. et al., 2019). IL-17A can induce osteoblast differentiation (Jo et al., 2018) and calcium-phosphate complexes can serve as potential catabolic mediators in cartilage (Jung et al., 2018). It has been reported that relaxin and cAMP are associated with anti-fibrogenic effects on OA synoviocytes (Qadri et al., 2018; Ko et al., 2019). Additionally, the activation of the p38MAPK pathway contributes to the overexpression of pro-inflammatory cytokines and chemokines and plays important roles in the progression of OA (Chen et al., 2018). Taken together, we speculated that exosomes may reduce inflammation of IL-1β-induced chondrocytes via the relaxin signaling pathway, IL-17 signaling pathway, TGF-beta signaling pathway, MAPK signaling pathway, TNF signaling pathway, cAMP signaling pathway, and calcium signaling pathway. Further studies should be performed to verify the role of these pathways in OA.

Exosomes contain mRNA, miRNA, lncRNA, and other signal substances, and can carry out information exchange between cells by targeting proximal cells in an autocrine and paracrine manner and targeting distal cells via the circulatory system (Colombo et al., 2014). LncRNAs, a type of non-coding RNA with a length of more than 200 bases, are regulators that do not encode proteins. MiRNAs are multifunctional non-coding RNAs with 22–25 bases and can regulate mRNA expression. Additionally, ceRNAs can sponge miRNAs to affect the expression of target mRNAs. Previous studies have shown that lncRNAs and miRNAs have regulatory effects on chondrocyte growth and inflammation, and play essential roles in the progression of OA (Swingler et al., 2019; Xie et al., 2020). Shen et al. (2018) found that lncRNA SNHG5 could serve as a ceRNA to compete with miR-26a and regulate SOX2 expression, thus participating in OA pathogenesis. In our study, by sequencing, we found a ceRNA network (lncRNA LYRM4-AS1—mRNA GRPR—miR-6515-5p) associated with the development of OA. A dual-luciferase reporter gene assay was used to confirm that LYRM4-AS1, miR-6515-5, and GRPR could interact with each other. The results of cell experiments showed that LYRM4-AS1 could mediate chondrocyte viability and apoptosis by regulating miR-6515-5p and GRPR. LYRM4-AS1 is related to integration hotspots in the human genome (Yang-Chun et al., 2020) and has not been explored in OA. GRPR, a gastrin-releasing peptide receptor, has been reported to be involved in the healing process in the synovium in patients with OA (Grimsholm et al., 2008). Xu et al. (2020) demonstrated that miR-6515-5p was highly expressed in P2 × 7R gene-modified stem cell-derived exosomes and reduced inflammation-mediated impairment of periodontal ligament stem cells through indirect binding to the GREM-1 protein. Together with our results, we speculate that exosomes isolated from human BMSCs may regulate chondrocyte growth by the LYRM4-AS1/GRPR/miR-6515-5p signal axis, reducing inflammation in OA.

However, this study has some limitations. First, the roles of exosomes and the LYRM4-AS1/GRPR/miR-6515-5p signal axis in OA need to be further investigated *in vivo*. In addition, experiments on the effects of relaxin signaling pathway, IL-17 signaling pathway, TGF-beta signaling pathway, MAPK signaling pathway, TNF signaling pathway, cAMP signaling pathway, and calcium signaling pathway in OA should be carried out. In conclusion, exosomes isolated from human BMSCs could exert a protective effect on OA, and the possible molecular protective mechanisms of exosomes in OA may be associated with MMP3, AKT, PTEN, and GRPR expression and the LYRM4-AS1/GRPR/miR-6515-5p signaling pathway. In addition, exosomes may alleviate OA inflammation through the relaxin signaling pathway, IL-17 signaling pathway, TGF-beta signaling pathway, MAPK signaling pathway, TNF signaling pathway, cAMP signaling pathway, and calcium signaling pathway. Our findings provide a theoretical basis for the application of exosomes in the treatment of OA and provide a basis for novel LYRM4-AS1/GRPR miR-6515-5p therapeutic targets and pathways for OA treatment.

## Data Availability Statement

The original contributions presented in the study are included in the article/Supplementary Material, further inquiries can be directed to the corresponding author/s.

## Author Contributions

XW and ZW designed the experiments. XW, ZL, YC, XC, and CC performed the experiments and analyzed the experimental results. ZW obtained the funding and supervised the experiments. XW drafted the manuscript and ZW revised the manuscript. All authors have reviewed and approved the submitted version of the manuscript.

## Conflict of Interest

The authors declare that the research was conducted in the absence of any commercial or financial relationships that could be construed as a potential conflict of interest.
