# Supplementary material for: Exosomes Isolated From Bone Marrow Mesenchymal Stem Cells Exert a Protective Effect on Osteoarthritis via lncRNA LYRM4-AS1-GRPR-miR-6515-5p
Source: Front Cell Dev Biol. 2021 May 28;9:644380. doi: 10.3389/fcell.2021.644380 (PMC8193855; doi:10.3389/fcell.2021.644380)
Supplement: Supplementary file 1 [file Data_Sheet_1.docx]

Supplementary Material

## Supplementary Figures


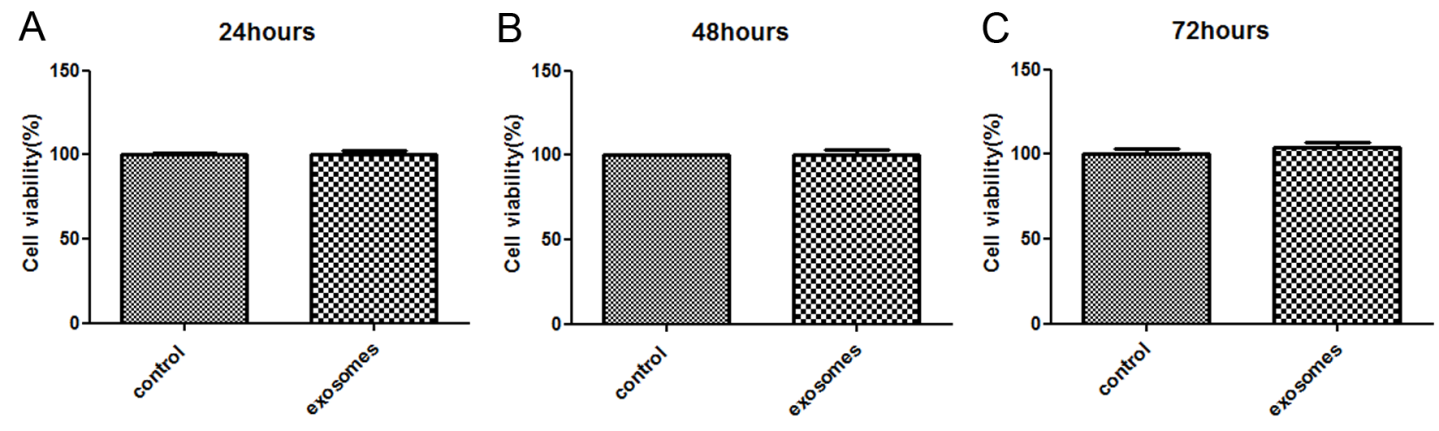


**Supplementary Figure 1.** Cell viability of chondrocytes treated with 20 μg/mL exosomes for 24 h (**A**), 48 h (**B**) and 72 h (**C**) using Cell Counting Kit-8 assay. n=3.


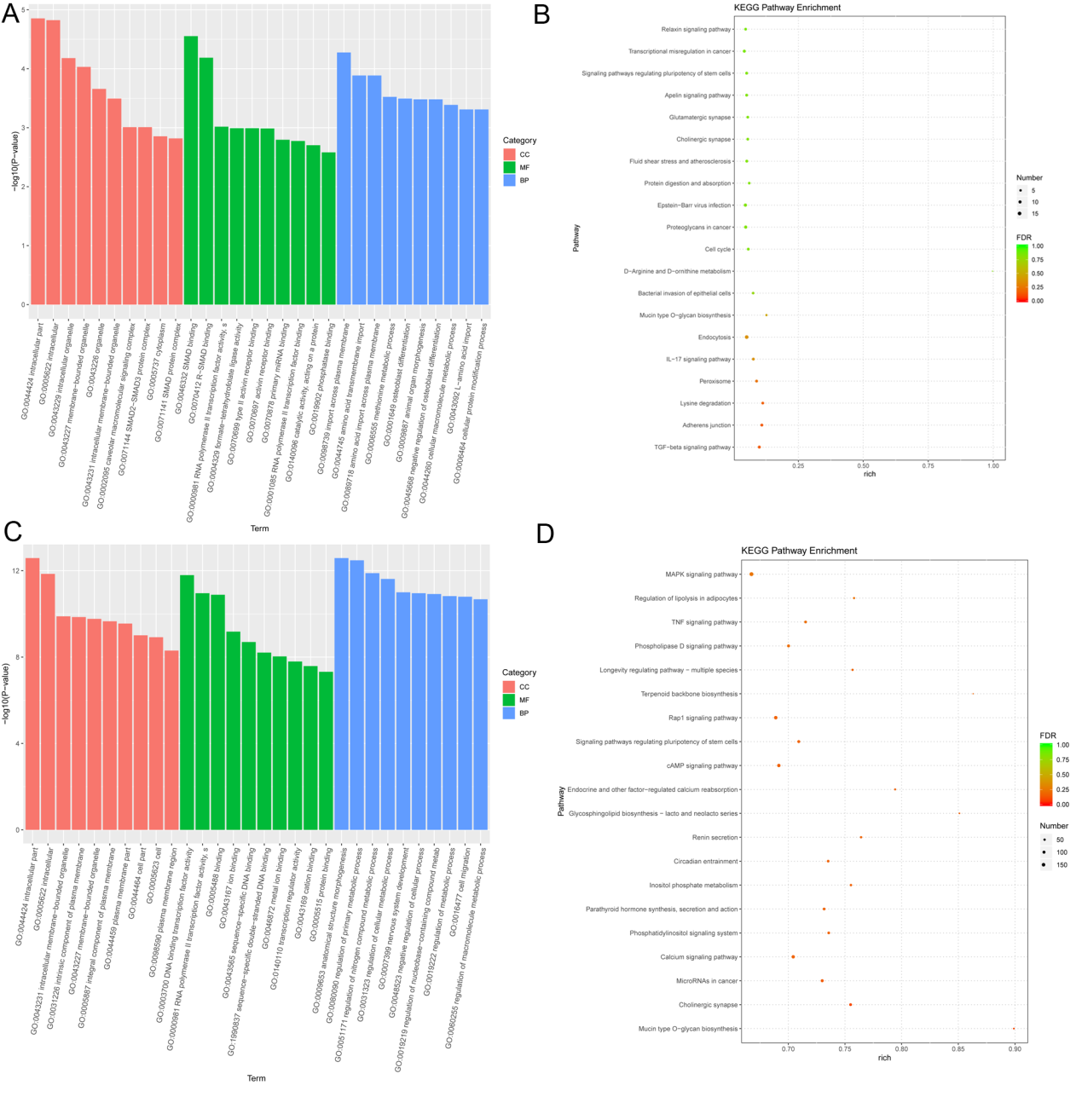


**Supplementary Figure 2.** Functional analyses of differentially expressed lncRNAs (DE-lncRNAs) and differentially expressed miRNAs (DE-miRNAs). (A) Gene Ontology (GO) terms analysis of DE-lncRNAs. (B) Kyoto Encyclopedia of Genes and Genomes (KEGG) pathways enrichment of DE-lncRNAs. (C) GO terms analysis of DE-miRNAs. (D) KEGG pathways enrichment of DE-miRNAs.
